# Different Barriers, Different Needs: A Qualitative Study on How Educational Level Shapes Barriers to Type 2 Diabetes Self-Management in Urban Pakistan

**DOI:** 10.7759/cureus.95477

**Published:** 2025-10-26

**Authors:** Shafat Khatoon, Muhammad A Miraj, Valeed B Mansoor, Qurat Ul Ain Alam, Syed Khurram Shaheer Kazmi, Farwa Rasheed, Abdul Mannan, Muhammad Aqeel

**Affiliations:** 1 Medicine, Shaheed Zulfiqar Ali Bhutto Medical University, Islamabad, PAK; 2 Medicine, Pakistan Institute of Medical Sciences, Islamabad, PAK; 3 Internal Medicine, Pakistan Institute of Medical Sciences, Islamabad, PAK; 4 Internal Medicine, Shaheed Zulfiqar Ali Bhutto Medical University, Islamabad, PAK; 5 General Medicine, Pakistan Institute of Medical Sciences, Islamabad, PAK

**Keywords:** cultural competency, diabetes education, educational level, self-care barriers, type 2 diabetes mellitus

## Abstract

Objective

This study aimed to explore how individuals with different educational levels in urban Pakistan experience barriers to type 2 diabetes mellitus (T2DM) self-care across key self-care dimensions.

Methods

A qualitative phenomenological study was conducted at the Diabetes Clinic of the Pakistan Institute of Medical Sciences, Islamabad. Forty-three adults diagnosed with T2DM were recruited and stratified by educational level (≥ high school, n = 17; < high school, n = 26). Semi-structured interviews were conducted, and transcripts were thematically analyzed to examine how educational level shapes barriers to T2DM self-care within Pakistan’s sociocultural context. Coding was performed iteratively until thematic saturation was reached, ensuring comprehensive representation of participant perspectives.

Results

Participants with higher education reported barriers such as time constraints due to demanding workloads and restrictive sociocultural expectations that limit engagement in public physical activity (e.g., walking) and structured meal planning. Many also reported receiving insufficient dietary guidance from healthcare providers tailored to their schedules and lifestyles. Predominant misconceptions among participants with lower education included equating household chores with adequate exercise and failing to recognize hypoglycemic symptoms. They also demonstrated limited understanding of dietary principles, such as believing that fruit juice is beneficial for individuals with T2DM. Notably, participants across both educational strata found it difficult to adhere to dietary restrictions in social settings, particularly during weddings and communal meals, where refusing food is culturally discouraged.

Conclusions

Barriers to T2DM self-care in urban Pakistan differ markedly by educational level. Improving T2DM self-care requires education-sensitive, community-based interventions tailored to the local sociocultural context. Higher educational attainment alone does not ensure adequate self-care, as cultural norms exert a strong influence that education alone cannot overcome. For individuals with lower education, self-care programs should prioritize tailored foundational dietary education to address fundamental knowledge gaps. For those with higher education, interventions should emphasize practical implementation strategies, including structured guidance on integrating self-care practices into demanding work routines and community-based initiatives that promote physical activity and challenge restrictive cultural norms. Such context-specific, stratified approaches may enhance T2DM self-care, improve glycemic control, and lead to better health outcomes.

## Introduction

Type 2 diabetes mellitus (T2DM) is a chronic metabolic disorder characterized by hyperglycemia resulting from impaired insulin action [1]. Globally, more than 500 million people live with T2DM, and this number is projected to increase substantially by 2050 [2]. The burden is disproportionately concentrated in low- and middle-income countries such as Pakistan, which account for more than 80% of global cases [2]. In Pakistan, T2DM affects over 30% of the population, with prevalence higher in urban than in rural settings; the number of patients with T2DM is projected to rise to 70 million by 2050 [2-4]. As this burden grows, it is crucial to identify effective strategies for managing T2DM.

Effective management of T2DM requires patients to learn and consistently apply appropriate self-care behaviors. Serious complications affecting vision, cardiovascular health, and kidney function are common in T2DM but can largely be prevented through effective self-care [5]. Key dimensions of self-care include physical activity, dietary control, medication adherence, blood glucose monitoring, hypoglycemia management, and appropriate healthcare utilization [6]. Among these, maintaining regular physical activity and dietary control are particularly challenging for many patients [5]. Evidence shows that patient-centered education programs can significantly improve T2DM self-care practices, particularly when interventions are tailored to the specific needs and contexts of target populations [7,8].

Self-care is fundamentally preventive, aiming to reduce the incidence and severity of T2DM-related complications. In Pakistan, quantitative studies have shown that higher educational levels are associated with better self-care practices [9,10]. However, these quantitative findings do not explain why education makes a difference, nor how contextual and cultural factors influence self-care behaviors across educational strata. A qualitative exploration is therefore needed to uncover the nuanced, context-specific barriers that quantitative data alone cannot capture.

Existing qualitative research in Pakistan has largely focused on rural populations, while urban contexts remain underexplored [11]. Moreover, urban Pakistan presents distinct challenges such as time pressures, sedentary lifestyles, and cultural obligations that may interact with educational attainment to influence self-care. No study has yet examined how barriers to T2DM self-management differ by educational level in this setting. Understanding these differences is essential not only for removing barriers but also for optimizing existing facilitators and identifying new ones.

Exploring differences by educational level will generate valuable insights into self-care practices within this population and inform the design of education-sensitive, culturally grounded interventions. Such interventions can avoid redundancy for those who already possess relevant knowledge while ensuring that educational content remains comprehensible and applicable for individuals with less foundational understanding.

The objective of this qualitative study is to explore differences in barriers to self-care across educational levels among adults with T2DM in urban Pakistan.

## Materials and methods

Study design and setting

This study employed a phenomenological qualitative design to examine how barriers to self-care among individuals with T2DM vary by educational level. In-depth interviews were conducted using a flexible, semi-structured guide that encouraged open-ended responses with patients attending the Diabetes Clinic at the Pakistan Institute of Medical Sciences, Islamabad.

Ethical approval

Ethical approval was granted by the Ethical Review Board of the Pakistan Institute of Medical Sciences (reference # F-5-2/2024(ERRB)/PIMS), in accordance with the principles of the Declaration of Helsinki. Written informed consent was obtained from all participants after they received written and verbal explanations of the study’s purpose and procedures. Confidentiality was safeguarded through the use of unique identifiers, and access to study data was restricted to members of the research team.

Study sample and data collection

A purposive sampling method was used to recruit participants to ensure inclusion of both educational strata and approximate gender parity across groups. All consenting participants visiting the Diabetes Clinic were eligible if they were Pakistani nationals aged ≥ 18 years, had a clinical diagnosis of T2DM for ≥ 3 years, and resided in an urban area of Islamabad. Individuals with other forms of diabetes, pregnant women, or those with severe comorbidities (e.g., advanced dementia and severe mental illness) were excluded.

The ≥ 3-year disease-duration criterion was selected to ensure participants had sufficient experiential familiarity with T2DM self-management and had encountered a range of barriers. Although this criterion may have biased the sample toward participants with greater disease experience, it enhanced the study’s ability to elicit nuanced and reflective accounts of self-care challenges.

Participants were divided into two educational strata: Group A: at or above high school (Grade 12 or equivalent) and Group B: below high school. Grade 12 was designated as the educational cut-off because, under Pakistan’s National Education Policy, completion of higher secondary education signifies eligibility for tertiary enrollment and generally corresponds to attainment of functional literacy necessary for comprehending written medical instructions and health-related information.

Interview content

Interviews were conducted in Urdu and audio-recorded until thematic saturation was reached [12]. A semi-structured interview guide was developed based on self-care dimensions identified in the literature and through consultations with T2DM experts to ensure coverage of relevant domains. Interviews took place in a vacant consultation room within the clinic to ensure participant privacy. Field notes were not taken because interactions were seated and comprehensively captured by audio recordings.

Data were collected according to the following self-care dimensions: physical activity, dietary control, medication adherence, blood glucose monitoring, hypoglycemia management, and healthcare utilization.

The interview guide was piloted with three patients to confirm cultural appropriateness and comprehensibility. Questions were adjusted as needed: some were expanded for clarity, and others were revised for wording. These pilot interviews were excluded from the final analysis. To elicit rich narratives, questions were structured to be open-ended.

Analysis

Author KM translated and transcribed all recordings into English. To ensure that the translations were precise, complete, and unbiased, author VBM carefully reviewed the final transcripts. Subsequently, two researchers (VBM and MAM) independently conducted coding using NVivo^®^, Version 12 (Released 2018; QSR International, Denver, CO, USA).

A hybrid deductive-inductive thematic analysis was employed: predefined self-care dimensions (e.g., physical activity and medication adherence) guided the initial coding framework, while distinct barriers within each domain were identified inductively from the data. Codes were iteratively organized into overarching thematic categories, and thematic saturation was considered reached when no new themes emerged, ensuring comprehensive representation of participants’ perspectives [13].

Any discrepancies in coding between the two investigators were resolved through a structured consensus process involving discussion, code comparison, and reflexive examination of interpretive assumptions. Divergent interpretations were reconciled by referring to the original transcripts, with arbitration by a third researcher (SK) to enhance analytical rigor and interpretive validity.

## Results

Forty-three patients, stratified by educational level, participated in the study. Group A (n = 17) comprised 10 males and seven females, while Group B (n = 26) comprised nine males and 17 females. Participants’ ages ranged from 31 to 87 years, and all reported Urdu as their first language. The characteristics of the study participants are summarized in Table 1.

**Table 1:** Characteristics of study participants (N = 43) ¹ Median (IQR) ² Mean ± SD ³ Frequency N (percentage %) Group A: educational level at or above high school; Group B: educational level below high school

Characteristic	Group A (frequency n = 17)	Group B (frequency n = 26)
Duration of T2DM (years)¹	10 (4-21)	9 (3-22)
Age (years)²	48.60 ± 11.90	54.70 ± 14.50
Gender³		
Male	10 (58.80%)	9 (34.60%)
Female	7 (41.20%)	17 (65.40%)
Employment status³		
Employed	13 (76.50%)	16 (61.50%)
Unemployed	4 (23.50%)	10 (38.50%)
Marital status³		
Single	2 (11.80%)	4 (15.40%)
Married	14 (82.40%)	19 (73.10%)
Widowed	1 (5.90%)	3 (11.50%)
Insulin usage³	5 (29.40%)	9 (34.60%)

The overrepresentation of females in Group B and males in Group A may have influenced the distribution of reported barriers, particularly in the domains of physical activity and dietary control, where gender roles and social expectations often intersect with educational level. Following thematic analysis, six overarching themes were identified. Table 2 presents the themes and corresponding subthemes for both groups.

**Table 2:** Thematic overview of differences in barriers to T2DM self-care according to educational level Group A: educational level at or above high school; Group B: educational level below high school

Diabetes self-care themes	Group A	Group B
Physical activity	Time and lifestyle constraints	Lack of awareness about benefits
	Cultural and social dynamics	Confusing everyday activity with exercise
Dietary control	Competing priorities	Nutritional awareness gaps
	Financial constraints	Cravings
	Social pressures	Social pressures
Medication adherence	Increased workload	Lack of medication understanding
		Forgetfulness
Blood sugar monitoring	Reliance on physical symptoms	Financial constraints
		Unawareness of target sugar levels
Healthcare usage	Lack of patient-centric communication	Reliance on informal networks
Hypoglycemia awareness	No reported barriers	Gaps in symptom knowledge
		Lack of awareness of emergency response measures

Theme 1: Physical activity barriers

Group A

Time and lifestyle constraints: Participants attributed their inconsistent physical activity to lifestyle-related factors such as busy schedules, lack of motivation, or insufficient time for exercise due to competing priorities.

*I don’t get time for exercise due to my work routine - *Group A; PA8; Male

Cultural and social dynamics: Participants reported that social obligations and cultural norms sometimes discouraged physical activity in daily life. While many engaged in regular exercise routines, others felt a lack of social support from their surrounding community.

*It feels awkward to walk in public areas because it’s not a common practice in our community* - Group A; PA16; Female

Group B

Lack of awareness about benefits: Most participants in this group were unaware of the crucial role that exercise plays in managing diabetes. Although some acknowledged the general importance of physical activity, they lacked specific knowledge about its benefits in controlling blood glucose levels, leading to inconsistent or minimal engagement in exercise routines. Moreover, the few participants who were aware of the role of exercise in diabetes management seldom allocated dedicated time for it, instead considering their daily activities as sufficient “exercise.”

*I didn't know I should exercise daily; I thought medicines were enough *- Group B; PB8; Female

Confusing everyday activity with exercise: Most participants in this group considered the physical exertion involved in their daily routines to be their form of “exercise.”

*I don’t do formal exercise, but I get some walking done while doing household work* - Group B; PB15; Female

Theme 2: Dietary control barriers

Group A

Competing priorities: Other responsibilities often took precedence over dietary control, with participants prioritizing medication adherence and the management of immediate health concerns. This led to inconsistent meal planning and reliance on convenient, less healthy food options during busy periods.

*I focus more on medications than maintaining a strict diet* - Group A; PA3; Male

*With work and family responsibilities, meal planning often takes a backseat* - Group A; PA14; Female

Financial constraints: Limited financial resources affected participants’ ability to access or prioritize healthier food options, often forcing them to make compromises.

*Healthy food is expensive, so I eat what’s made at home, even if it’s not always good for my sugar levels* - Group A; PA4; Male

*We try to cut down expenses and not compromise on health. So, we try to bring better food at home, but it’s tough* - Group A; PA16; Female

*The doctor told me not to eat white flour, but it’s hard to find affordable alternatives* - Group A;​​​​​​​ ​​​​​​​PA11; Male

Social pressures: Cultural norms during social gatherings posed challenges, as participants found it difficult to adhere to dietary restrictions in group settings.

*When I attend a wedding or function, I eat whatever is served, even if it’s not good for my sugar control *- Group A;​​​​​​​ ​​​​​​​PA1; Male

Group B

Gaps in nutritional awareness: Participants in Group B demonstrated a marked lack of knowledge about proper dietary practices, leading to frequent dietary errors. Many were unaware of the glycemic impact of common foods such as potatoes, white bread, and dates and continued to consume them. Several did not limit their fruit intake and reported difficulty interpreting dietary advice from healthcare providers, describing it as too vague.

*I thought all fruits were okay, but some make my sugar worse* - Group B; PB13; Male

*I thought drinking fruit juice was healthy, but my sugar levels kept going up* - Group B; PB22; Female

Cravings: Many participants struggled to resist cravings for sweet or high-carbohydrate foods. Despite being aware of their adverse effects on blood sugar levels, they often found it difficult to control personal preferences, leading to dietary noncompliance.

*I know I shouldn’t eat sweets, but I love them, and sometimes I can’t resist -* Group B;​​​​​​​ ​​​​​​​PB21; Male

Social pressures: Social gatherings, family traditions, and cultural practices often involve foods incompatible with diabetes management. Participants reported difficulties adhering to dietary restrictions during such events.

*When I visit relatives, they insist I eat what they serve, even if it’s not good for my diabetes* - Group B; PB17; Female

*I can’t resist having some dessert during celebrations* - Group B; PB9; Male

Theme 3: Medication adherence barriers

Group A

Increased workload: Adherence was affected by external factors such as workload, forgetfulness, and a dislike of taking medications. Some participants occasionally skipped doses, perceiving their condition as manageable even without strict compliance.

*Sometimes it gets missed due to a hectic workload, but I take medication as soon as I remember* - Group A; PA15; Male

Group B

Lack of understanding about medication: Some participants had limited knowledge about their prescribed medications, including their purpose, correct dosage, and timing. This lack of understanding often led to misuse or inconsistent adherence.

*I don’t know which pill is for diabetes and which is for blood pressure *- Group B; PB25; Male

Forgetfulness: Participants frequently identified forgetfulness as a major barrier to medication adherence, particularly during busy mornings or stressful periods. Age-related memory decline or other health conditions also contributed to missed doses.

*I forget to take my medicine before breakfast sometimes *- Group B; PB17; Female

Theme 4: Blood sugar monitoring barriers

Group A

Reliance on physical symptoms: A few participants in this group relied on physical symptoms such as dizziness or weakness rather than regular blood glucose monitoring. This approach increased the risk of missing asymptomatic fluctuations. Participants noted that sensations like fatigue or headaches often prompted them to check their blood sugar levels.

*I don’t check much now unless I feel symptoms like tiredness or pain *- Group A;​​​​​​​ ​​​​​​​PA6; Male

Group B

Financial constraints: Financial constraints prevented many participants from obtaining glucometers. These individuals relied on clinic visits for blood sugar monitoring, which often limited the frequency of checks and delayed adjustments to their diabetes management plans. Some of those who did have glucometers were not able to monitor regularly due to the inability to afford strips.

*I rely on the clinic near my home to check my sugar because I don’t have a machine at home *- Group B;​​​​​​​ ​​​​​​​PB18; Female

*I can’t always afford the test strips, so I only check my blood sugar when I think it’s really needed *- Group B;​​​​​​​​​​​​​​ PB16; Male

Unawareness of target sugar levels: A significant barrier to regular monitoring in this group was participants’ lack of knowledge about their ideal fasting and postprandial blood sugar levels. Many expressed confusion about why monitoring was important or how to interpret the results.

I don’t understand what these numbers mean after I check my sugar - Group B; PB20; Male

Theme 5: Healthcare usage barriers

Group A

Lack of patient-centric communication: Communication issues, such as a lack of detailed explanations about treatment and dietary plans, led to confusion and dissatisfaction among participants.

*I wish doctors would explain a bit about medications instead of just handing over prescriptions -* Group A;​​​​​​​​​​​​​​ PA13; Female

No one (health provider) gave me a specific diet plan; they just told me to avoid sweet and fatty foods - Group A;​​​​​​​​​​​​​​ ​​​​​​​PA17; Male

Group B

Reliance on informal networks: Many participants reported turning to friends or relatives for information in the absence of continuing professional healthcare, mostly due to financial constraints, as the majority of people pay for all their health expenditure out of pocket. Some also rely on neighbors for dietary advice and information regarding blood glucose target ranges.

*I rely on advice from relatives who have similar conditions* - Group B;​​​​​​​​​​​​​​ ​​​​​​​PB2; Female

Theme 6: Hypoglycemia awareness barriers

Group A

No barriers were observed in this group for this self-care dimension.

Group B

Gaps in symptom knowledge: The majority of participants could not identify the early signs of hypoglycemia, such as fatigue, dizziness, shaking, and sweating.

*Sometimes, in the afternoon, I feel very cold. So I go out and sit in the sun* - Group B;​​​​​​​​​​​​​​ ​​​​​​​PB11; Female

Lack of awareness of emergency response measures: Most participants lacked a clear plan for emergency management in case of a hypoglycemic episode. Some expressed relying on their family members to address the situation if it arises again (based on prior such experiences).

*I’m not sure how to manage low sugar on my own, so I depend on my family to help* - Group B;​​​​​​​​​​​​​​ ​​​​​​​PB6; Female

## Discussion

This study reveals that distinct barriers exist to T2DM self-care according to educational level. Patients with lower educational levels face foundational knowledge gaps and affordability issues, while patients with higher educational levels struggle with implementing knowledge due to time pressures or inhibiting cultural norms.

Previous qualitative studies have described barriers to T2DM self-care in Pakistan, but none have stratified those by educational level. Our study shows that educational level shapes distinct barriers across T2DM self-care dimensions, providing granular evidence to inform differentiated intervention design. While conducted in urban Pakistan, these themes may resonate with other South Asian populations, as suggested by studies grouping Pakistani and Indian communities based on similar T2DM-related self-care beliefs and challenges [14,15].

It is important to recognize that prevailing self-care models are a Western cultural construct. Evidence indicates that culturally adapted interventions for South Asians are not only more acceptable but also more effective; systematic reviews have shown that such interventions achieve significantly greater improvements in HbA1c levels and patient knowledge scores compared to non-culturally adapted interventions [16]. This shows that self-care interventions in South Asian populations, including Pakistanis, should be designed and delivered in a culturally responsive, context-aware manner to maximize impact [8,17].

Evidence from a UK-based randomized controlled trial of British Pakistani women further illustrates this need. While a culturally appropriate, structured health education intervention improved knowledge and glycemic control overall, women with lower educational levels did not show the same gains in either knowledge or glycemic outcomes [18]. Hence, even culturally adapted interventions may lose effectiveness if they fail to account for differences in educational level.

Our findings reveal distinct patterns in how educational level shapes barriers to engaging in physical activity. Participants with higher education cited time constraints and cultural norms discouraging outdoor exercise as major obstacles, despite being aware of its health benefits. Similar cultural barriers have been reported by Jepson et al. and Naeem [19,20]. In contrast, those with lower education confused routine daily chores, such as housework or short walks to the office, with adequate exercise, reflecting a gap in understanding of physical activity as it pertains to T2DM self-care. In contrast to the barriers faced by rural residents reported by Ansari et al., no participant in our urban study cited a lack of facilities as an obstacle [21].

Cultural norms in South Asian contexts particularly restrict women’s engagement in outdoor physical activity, a gender disparity also reported by Arsh et al. [22]. Studies of Pakistani women in the UK and the US have identified similar cultural barriers, showing that these challenges extend beyond urban Pakistan to diaspora communities and that there is a need for culturally sensitive, context-specific strategies that can be adapted to individual routines and social settings [23].

Educational level also shaped participants’ barriers to dietary self-care in distinct ways. Those with higher education understood the principles of healthy eating for T2DM but reported challenges in implementing this knowledge, citing the higher cost of healthier food options and a lack of time or skills to prepare them. In contrast, participants with lower education faced more fundamental knowledge gaps, including unawareness of which foodstuffs to avoid, such as dates, fruit juice, and white bread, and frequently reported difficulty in managing cravings. Omama et al. noted similar gaps in essential dietary knowledge [24]. Likewise, Bukhsh et al. reported difficulties in managing cravings, which led to high sugar intake [25]. Consumption of sweet foods had also been reported by Lawton et al. as a significant challenge [26].

Both groups described a shared barrier stemming from expectations around social gatherings and communal events, such as weddings, that made refusing unsuitable foods difficult. In Pakistani culture, refusing food offered by the host is considered a sign of disrespect [26]. With alternatives unavailable, patients are resigned to eating unhealthy food, even in informal settings such as visiting relatives. Similar patterns have been reported in other studies, which also emphasize the importance of delivering dietary education, advice, and support to entire families rather than solely to the person with T2DM [24,26]. Given that the joint family system is widely prevalent in Pakistan and that responsibility for food purchasing and preparation is often shared among household members, interventions should incorporate family-oriented and community-level elements to improve overall awareness of healthy eating and support practical behavior changes [26].

Managing T2DM effectively requires consistent adherence to prescribed medications, yet participants in this study described multiple barriers that impeded this critical aspect of self-care. Among those with higher education, missed doses were often attributed to demanding work schedules or travel, during which they did not bring their medications. These findings are consistent with those reported by Bukhsh et al. [25]. Some participants believed that strict adherence was not essential for adequate self-care and felt that occasionally skipping doses was acceptable. In contrast, participants with lower education levels frequently cited forgetfulness as their main challenge to adherence. None reported using reminder systems or simple visual cues, such as placing medicines in prominent locations. Moreover, many in this group struggled to distinguish between diabetes medications and those for other conditions, depending on family members for assistance. Similar findings were observed by Patel et al. among Pakistanis [15].

According to the conceptual model of medication adherence barriers by Naqvi et al., forgetfulness is associated with inadequate knowledge about medications [27]. Cost further complicated adherence in this group, reflecting unintentional non-adherence driven by financial constraints rather than a lack of willingness. These findings highlight the importance of ensuring that patients fully understand their treatment regimens through clear, supportive communication from healthcare providers.

Financial barriers were especially pronounced among participants with lower education levels. Many could not afford glucometers for regular blood sugar monitoring, and those who did often struggled to buy test strips, leading to sporadic checks at nearby health facilities instead. Low health literacy compounded these challenges, as participants were often unaware of target blood sugar ranges, limiting their ability to manage their T2DM effectively. Addressing these affordability issues is essential to improving equitable access to self-care resources and helping reduce the incidence of preventable complications.

In the higher education group, barriers to adequate monitoring were different. Many participants relied on physical sensations to gauge blood sugar levels instead of using readings, placing them at risk for unrecognized fluctuations that could turn deadly without warning. Participants’ experiences with healthcare providers varied by educational level, revealing important barriers to effective T2DM self-care. Those with higher education often reported dissatisfaction with provider interactions, describing self-care instructions, particularly dietary guidance, as vague or insufficiently detailed. As a result, they frequently turned to online resources to supplement the information provided. This contrasts with the findings of Bukhsh et al., who identified provider education as a facilitator for urban T2DM patients in Pakistan [25]. A similar experience of dissatisfaction has emerged from the findings of Ansari et al. [21].

In contrast, participants with lower education levels were more likely to rely on advice from family and friends. This tendency was shaped by perceptions of providers as unhelpful and resistant to questions and by the additional burden of out-of-pocket healthcare expenses. Such barriers contributed to inadequate knowledge about medications and their correct usage. This informal network, comprising family, friends, acquaintances, and extended relatives, often perpetuates traditional folk knowledge, influencing food choices and the use of complementary and alternative medicine (CAM) [24].

Despite its importance, Pakistani health professionals, socialized within a colonial Western medical framework, often undervalue local traditions and health-seeking behaviors [24]. Early evidence from urban Pakistan indicates that more than two-thirds of T2DM patients use some form of CAM [28]. A major contributing factor driving patients toward CAM, including herbal remedies, homeopathic medicine, and spiritual healing, is dissatisfaction with conventional healthcare providers, along with lower associated costs [28]. Improving provider-patient communication, ensuring patient understanding, and fostering open, supportive communication are essential strategies for supporting effective T2DM self-care across education levels [29]. This is particularly important for patients with lower educational levels, who may be less likely to seek additional information online despite having the means to do so.

Hypoglycemia is an acute complication of T2DM and can be lethal if not managed properly. Neurological manifestations, such as impaired concentration and seizures, pose significant risks to patient safety and have been associated with an increased incidence of traffic accidents [30].

In our study, hypoglycemia management emerged as a particular challenge for participants with lower education levels, who often lacked awareness of symptoms like cold sweats or dizziness, even when they occurred. These hypoglycemic episodes were exacerbated during the month of Ramadan, when fasting routines increased the risk of a pattern also noted in other studies [31]. Participants also described inadequate coping strategies, such as sitting in the sun to counter cold sweats, and had limited knowledge of appropriate emergency responses. Targeted hypoglycemia awareness sessions could be a valuable intervention to improve symptom recognition and safe management of hypoglycemic episodes in this group.

Implications

Findings of our study have practical implications for designing interventions tailored to individual patient needs, which also address the education-stratified barriers identified in this study. They also offer valuable guidance for strengthening Pakistan’s healthcare system through culturally adapted approaches that engage T2DM patients, their families, and providers collaboratively.

For patients with lower educational levels, T2DM self-care education should prioritize clear, accessible, and culturally appropriate content that addresses foundational knowledge gaps about diet, exercise, and medication.

In contrast, for patients with higher education, interventions should focus on supporting habit formation through behavioral change strategies to help patients consistently apply existing knowledge despite time constraints and competing demands. The findings of this study can inform the design of behavior change interventions based on validated models such as COM-B.

Community-based interventions are essential to promote culturally acceptable forms of physical activity, including flexible indoor or home-based options that address social norms discouraging outdoor exercise, particularly among women. Evidence suggests that group-based physical activities designed for South Asian communities achieve better adherence rates and are perceived as more sustainable. Dietary interventions should similarly move beyond focusing solely on individual knowledge to adopt family-centered and community-based approaches that consider shared decision-making around food and cultural expectations around hospitality.

Healthcare providers should be attentive to family hierarchies to identify key influencers of meal choices and involve them directly in education. Practical strategies could include counselling family decision-makers during clinic visits and offering culturally appropriate guidance on healthier recipes, especially to women who typically oversee cooking in Pakistani households [24]. Such education could be delivered through T2DM-focused cookbooks or culturally responsive cooking shows. Counselling by health providers should be tailored to patients’ educational levels and should include techniques such as teach-back to confirm understanding, particularly for those with lower education.

At the policy level, financial inclusion measures could improve access to essential self-monitoring tools, such as glucometers and medications, for lower-income, less-educated populations. Digital literacy initiatives can also leverage widespread smartphone availability to support health information-seeking among patients with limited formal schooling and provide detailed, tailored information to those with higher educational levels.

Ultimately, coordinated efforts across patient, family, community, provider, and policy levels will be essential for improving T2DM self-care and consequently health outcomes for T2DM patients in Pakistan.

Future research could explore the development of culturally acceptable and gender-sensitive physical activity options for South Asian populations, with an emphasis on community-based interventions aimed at shifting social norms that discourage outdoor exercise. Studies could also investigate family-centered strategies to promote dietary change, given the strong influence of shared meals and social gatherings on food choices. Research could also examine emerging technologies such as wearable devices that use AI to predict blood glucose levels, which could be less invasive and potentially lower glucose monitoring costs [32].

Strengths and limitations

A key strength of this study lies in the use of in-depth, local-language interviews conducted until thematic saturation was reached, ensuring that participants’ perspectives were authentically captured and deeply explored. By stratifying barriers according to educational level, a variable often overlooked in T2DM self-care research, this study provides nuanced, actionable insights that can inform the design of education-sensitive interventions in Pakistan.

However, several limitations must be acknowledged. As participants were recruited from a single urban T2DM clinic in Islamabad, the findings may limit generalizability. Women were somewhat overrepresented in the lower-education group, which could partially confound the observed education-related differences in self-care behaviors. The reliance on self-reported data introduces the potential for recall and social desirability biases, although measures were taken to foster openness and trust during interviews.

Moreover, while the study examined education as a key stratifying variable, it did not separately analyze the roles of income, occupation, or employment status, which may interact with education and gender to shape self-care opportunities, a limitation that underscores the need for an equity and intersectionality lens. Future research should therefore quantify the relative influence of education alongside socioeconomic and gender-related factors, include participants from rural cohorts, and explore gender-specific educational barriers to refine and broaden the applicability of targeted diabetes self-care interventions.

## Conclusions

Educational level markedly shapes barriers to T2DM self-care in urban Pakistan. Patients with lower education face fundamental gaps in knowledge, affordability constraints, and reliance on informal networks that often perpetuate misinformation. Those with higher education struggle with time pressures, restrictive sociocultural norms, and applying existing knowledge about diet and physical activity. Higher educational level alone is not enough for adequate T2DM self-care. These findings highlight the need for education-sensitive, culturally adapted interventions that move beyond individual-level education and engage family, community, and health system dynamics. By tailoring the content, delivery, and support strategies of self-care interventions to patients’ educational levels and sociocultural contexts, practitioners can enhance self-care practices and ultimately improve health outcomes among individuals with T2DM in Pakistan.

## References

[1] Chatterjee S, Khunti K, Davies MJ (2017). Type 2 diabetes. Lancet.

[2] (2023). Global, regional, and national burden of diabetes from 1990 to 2021, with projections of prevalence to 2050: a systematic analysis for the Global Burden of Disease Study 2021. Lancet.

[3] Lin X, Xu Y, Pan X (2020). Global, regional, and national burden and trend of diabetes in 195 countries and territories: an analysis from 1990 to 2025. Sci Rep.

[4] Basit A, Fawwad A, Qureshi H, Shera AS (2018). Prevalence of diabetes, pre-diabetes and associated risk factors: second National Diabetes Survey of Pakistan (NDSP), 2016-2017. BMJ Open.

[5] Ahola AJ, Groop PH (2013). Barriers to self-management of diabetes. Diabet Med.

[6] Ortz CL, Duncan MS, Leshi O, Burrows WB, Smalls BL (2025). Influence of perceived health provider communication, diabetes duration and age at diagnosis with confidence in diabetes self-care. BMJ Open Diabetes Res Care.

[7] Sidani S, Ibrahim S, Lok J, Fan L, Fox M (2018). Implementing the integrated strategy for the cultural adaptation of evidence-based interventions: an illustration. Can J Nurs Res.

[8] Rahim E, Rahim FO, Anzaar HF, Lalwani P, Jain B, Desai A, Palakodeti S (2024). Culturally tailored strategies to enhance type 2 diabetes care for South Asians in the United States. J Gen Intern Med.

[9] Bukhsh A, Khan TM, Sarfraz Nawaz M, Sajjad Ahmed H, Chan KG, Goh BH (2019). Association of diabetes knowledge with glycemic control and self-care practices among Pakistani people with type 2 diabetes mellitus. Diabetes Metab Syndr Obes.

[10] Gillani AH, Amirul Islam FM, Hayat K (2018). Knowledge, attitudes and practices regarding diabetes in the general population: a cross-sectional study from Pakistan. Int J Environ Res Public Health.

[11] Ansari RM, Harris MF, Hosseinzadeh H (2022). Experiences of diabetes self-management: a focus group study among the middle-aged population of rural Pakistan with type 2 diabetes. Diabetology.

[12] Mason M (2010). Sample size and saturation in PhD studies using qualitative interviews. Forum Qual Soc Res.

[13] Braun V, Clarke V (2006). Using thematic analysis in psychology. Qual Res Psych.

[14] Kumar K, Greenfield S, Raza K, Gill P, Stack R (2016). Understanding adherence-related beliefs about medicine amongst patients of South Asian origin with diabetes and cardiovascular disease patients: a qualitative synthesis. BMC Endocr Disord.

[15] Patel NR, Chew-Graham C, Bundy C, Kennedy A, Blickem C, Reeves D (2015). Illness beliefs and the sociocultural context of diabetes self-management in British South Asians: a mixed methods study. BMC Fam Pract.

[16] Bhurji N, Javer J, Gasevic D, Khan NA (2016). Improving management of type 2 diabetes in South Asian patients: a systematic review of intervention studies. BMJ Open.

[17] Bellary S, O’Hare JP, Raymond NT (2008). Enhanced diabetes care to patients of South Asian ethnic origin (the United Kingdom Asian Diabetes Study): a cluster randomised controlled trial. Lancet.

[18] Hawthorne K (2001). Effect of culturally appropriate health education on glycaemic control and knowledge of diabetes in British Pakistani women with type 2 diabetes mellitus. Health Educ Res.

[19] Jepson R, Harris FM, Bowes A, Robertson R, Avan G, Sheikh A (2012). Physical activity in South Asians: an in-depth qualitative study to explore motivations and facilitators. PLoS One.

[20] Naeem AG (2003). The role of culture and religion in the management of diabetes: a study of Kashmiri men in Leeds. J R Soc Promot Health.

[21] Ansari RM, Hosseinzadeh H, Harris M (2019). Self-management experiences among middle-aged population of rural area of Pakistan with type 2 diabetes: a qualitative analysis. Clin Epidemiol Glob Health.

[22] Arsh A, Afaq S, Carswell C (2023). Barriers & facilitators to physical activity in people with depression and type 2 diabetes mellitus in Pakistan: a qualitative study to explore perspectives of patient participants, carers and healthcare staff. Ment Health Phys Act.

[23] Natesan A, Nimbal VC, Ivey SL, Wang EJ, Madsen KA, Palaniappan LP (2015). Engaging South Asian women with type 2 diabetes in a culturally relevant exercise intervention: a randomized controlled trial. BMJ Open Diabetes Res Care.

[24] Tariq O, Rosten C, Huber J (2024). Cultural influences on making nutritional adjustments in type 2 diabetes in Pakistan: The perspectives of people living with diabetes and their family members. Qual Health Res.

[25] Bukhsh A, Goh BH, Zimbudzi E, Lo C, Zoungas S, Chan KG, Khan TM (2020). Type 2 diabetes patients’ perspectives, experiences, and barriers toward diabetes-related self-care: A qualitative study from Pakistan. Front Endocrinol (Lausanne).

[26] Lawton J, Ahmad N, Hanna L, Douglas M, Bains H, Hallowell N (2008). 'We should change ourselves, but we can't': accounts of food and eating practices amongst British Pakistanis and Indians with type 2 diabetes. Ethn Health.

[27] Naqvi AA, Hassali MA, Aftab MT, Nadir MN (2019). A qualitative study investigating perceived barriers to medication adherence in chronic illness patients of Karachi, Pakistan. J Pak Med Assoc.

[28] Siddiqui F, Hewitt C, Jennings H, Coales K, Mazhar L, Boeckmann M, Siddiqi N (2024). Self-management of chronic, non-communicable diseases in South Asian settings: a systematic mixed-studies review. PLOS Glob Public Health.

[29] Adu MD, Malabu UH, Malau-Aduli AE, Malau-Aduli BS (2019). Enablers and barriers to effective diabetes self-management: a multi-national investigation. PLoS One.

[30] LaManna J, Litchman ML, Dickinson JK (2019). Diabetes education impact on hypoglycemia outcomes: a systematic review of evidence and gaps in the literature. Diabetes Educ.

[31] Ahmedani MY, Alvi SF, Haque MS, Fawwad A, Basit A (2014). Implementation of Ramadan-specific diabetes management recommendations: a multi-centered prospective study from Pakistan. J Diabetes Metab Disord.

[32] Ahmed A, Aziz S, Abd-Alrazaq A, Farooq F, Househ M, Sheikh J (2023). The effectiveness of wearable devices using artificial intelligence for blood glucose level forecasting or prediction: systematic review. J Med Internet Res.

